# Recent Progress in Development and Application of DNA, Protein, Peptide, Glycan, Antibody, and Aptamer Microarrays

**DOI:** 10.3390/biom13040602

**Published:** 2023-03-27

**Authors:** G. M. Aparna, Kishore K. R. Tetala

**Affiliations:** Centre for Bioseparation Technology (CBST), Vellore Institute of Technology (VIT), Vellore 632014, Tamilnadu, India; aparnagm96@gmail.com

**Keywords:** microarray, DNA, protein, peptide, glycan, antibody, aptamer

## Abstract

Microarrays are one of the trailblazing technologies of the last two decades and have displayed their importance in all the associated fields of biology. They are widely explored to screen, identify, and gain insights on the characteristics traits of biomolecules (individually or in complex solutions). A wide variety of biomolecule-based microarrays (DNA microarrays, protein microarrays, glycan microarrays, antibody microarrays, peptide microarrays, and aptamer microarrays) are either commercially available or fabricated in-house by researchers to explore diverse substrates, surface coating, immobilization techniques, and detection strategies. The aim of this review is to explore the development of biomolecule-based microarray applications since 2018 onwards. Here, we have covered a different array of printing strategies, substrate surface modification, biomolecule immobilization strategies, detection techniques, and biomolecule-based microarray applications. The period of 2018–2022 focused on using biomolecule-based microarrays for the identification of biomarkers, detection of viruses, differentiation of multiple pathogens, etc. A few potential future applications of microarrays could be for personalized medicine, vaccine candidate screening, toxin screening, pathogen identification, and posttranslational modifications.

## 1. Introduction

Studies on the cellular activities and individual functions of biological entities, such as proteins, DNA, and RNA, are of constant interest to biologists [1]. The need for accurate and rapid detection with the use of low sample volumes of these biological compounds in understanding disease progression, microbial pathogen detection, etc., has led to the development of microarray-based analysis techniques [1]. The basis of this concept started in 1989 and 1990 with the introduction of the “ambient analyte immunoassay” [2,3,4], which was materialized with the development of DNA microarrays [5,6]. With the scientific evidence that genetic information alone is not sufficient for having a deeper understanding of cellular networks [7,8], researchers started to develop protein-/antibody-based microarray systems [9,10].

A typical microarray chip involves the use of a flat substrate (glass, polymer, silica, plastics, or nitrocellulose) that is embedded with a large variety of molecules as microspots that have specific recognition of and potential for the target analyte(s) of interest [11,12]. The choice of substrate depends on its biocompatibility, ease of surface functionalization for probe attachment in favorable orientations, and low background or nonspecific binding tendencies [13]. Depending on the immobilized biomolecule, microarrays are categorized as DNA microarrays, protein microarrays, glycan microarrays, peptide microarrays, antibody microarrays, or aptamer microarrays. The detection of interaction is performed via the use of label or label-free methods that help in securing both quantitative and qualitative information [14]. The obtained data can then be analyzed in terms of quality control, normalization, and statistical analysis either using a specific software (SNOMAD, Nexus Expression, Goober, and FIGS) designed for the analysis or via certain databases, which act as archives of publicly available data [15].

The advantages of microarray technology are high-throughput sample screening, the multiplexing of several hundreds of molecules on a single chip, a low sample requirement, the study of diverse experimental parameters, data analyses, and obtaining insights on molecular interactions even at low sample concentrations [16,17]. However, their major limitations are a high cost for a single experiment, a large number of probe designs based on sequences of low specificity, and the high sensitivity of the experimental setup to variations in hybridization temperatures [18,19]. Over the years, new innovations and technological advancements were made to overcome the limitations of microarrays. These improvements were a direct result of transforming ideas on surface modification chemistries, biomolecule immobilization approaches, and a variety of substrate materials with new properties. The wide applications of microarrays include disease differentiation and detection studies, genomic analyses, expression analyses, proteomics, drug–target identification, vaccine candidate identification, host–pathogen interactions, and environmental monitoring [16].

Over the years, from time to time, various review articles or book chapters have been published on both the technological advancements and applications of microarrays in various fields. In the case of biomolecule-based microarrays, the focus of those research articles was majorly restricted to an individual array, i.e., a DNA microarray [20,21,22,23], protein microarray [24,25,26], glycan microarray [27,28,29], peptide microarray [30,31,32], or antibody microarray [13,33,34]. The truncation of the microarray technology reviews based on individual biomolecules has its own advantages (serves the purpose of those specialized in one particular subdomain or a more focused area). However, the disadvantage (for specialists in other subdomains) might be losing track of exciting developments in other subdomains. Additionally, in these reviews, the discussions on biomolecule immobilization strategies were not discussed in detail. Considering, the spectrum of fields (chemistry, engineering, and biology) involved in microarray development stages and applications, a review article that touches all these aspects is important.

In this review, we aim to cover the five major biomolecule (DNA, protein, glycan, antibody, and aptamer)-based microarray chip developments and their applications since 2018 onwards. There are also other microarrays, such as tissue microarrays, cellular microarrays, phenotype microarrays, and small-molecule-based microarrays. However, in this review, we have restricted ourselves to only the above-mentioned five biomolecules. We have categorized the review into three parts: microarray chip preparation, applications, and conclusions with future directions. In the first part, the emphasis was laid on the technological developments made in terms of microchip fabrication strategies, biomolecule (DNA, protein, peptide, glycan, antibody, and aptamer) immobilization chemistries, detection methods, and new detection approaches. In the second part, the applications of these biomolecule-based microarrays were discussed. Here, the focus was also laid on the microarrays’ selectivity and specificity in comparison to the existing methods. In the third and final part, a general conclusion on various aspects was highlighted along with our views on the future trends of microarrays.

## 2. Microarray Fabrication, Biomolecule Immobilization, and Detection Techniques

A microarray chip is a product that is ready to be used for its intended application. The workflow to prepare this microarray chip includes three stages: chip fabrication, substrate surface modification, and biomolecule immobilization. The microchip with the desired biomolecules is then treated with biological fluids (plasma, urine, saliva, etc.) to capture the target molecules of interest. The detection of the captured molecules can be monitored in two ways: the use of labels (fluorescence tags) or label-free methods (mass spectrometry, surface plasmon resonance (SPR), reflectometric interference spectroscopy (RIfS), and oblique-incidence reflectivity difference (OIRD)). This section covers the developments in microarray fabrication, biomolecule immobilization, and detection techniques.

### 2.1. Fabrication Strategies

During the initial years of development, polyvinylidene fluoride membranes were used as supporting substrates because of their optimized density as membranes [35]. In the following years of advancement, various materials, such as glass, polymers, plastics, nitrocellulose, porous gel slides, and silicon slides, came into the foray [13,15,36]. The naked substrate surface was not suitable for microarray application due to lack of active functional groups for biomolecule (DNA, antibody, protein, aptamer, and glycan) immobilization, high nonspecific biomolecule adhesion, and high background noise during detection [13,37,38,39]. Hence, depending on the application and the substrate used, they undergo various chemical processes (chemical treatments for glass and aminolysis reactions for polymers such as polymethylmethacrylate (PMMA)). Depending on the reagents used for surface treatment, various active functional groups, such as aldehydes (-C=O), amines (-NH_2_), thiols (-SH), and epoxides, are generated on the substrate. Recently, hollow silica nanoparticles were coated on cyclic olefin copolymer sheets and were used as substrates for microarray development [40]. This array has displayed a good sensitivity for the chosen model molecule, i.e., IgG. Similarly, self-assembled monolayers with different functional groups (epoxides and amines) were grafted on glass and silicon chips to fabricate protein microarrays [41]. The fluorescence-labeled antigen of the HSV-1 virus was used as a model molecule to study protein immobilization.

In general, microarray fabrication is performed via photolithography, mechanical microspotting, and inkjet printing [20,42,43,44,45]. The fabrication of DNA microarrays is also performed via other approaches, such as oligonucleotide-based microarrays, electronic-based microarrays, and suspension-bead microarrays [20]. Since 2018, only Thanthringe-Don et al. [46] have explored the electronic-based microarray platform (Nanochip 400, Nanogen) for DNA microarray fabrication. Readers are referred to [45] for more detailed information on microarray performance parameters, such as array geometry, spot density, spot characteristics, and background.

***Photolithography*** involves the use of a photomask with a defined shape and size on the substrate followed by the exposure of the unmasked region to UV light to remove the photolabile groups on the surface [47]. For DNA/RNA microarrays, a designated nucleotide is immobilized followed by the addition of a blocking agent. This cycle of masking, UV exposure, nucleotide immobilization, and the addition of a blocking agent is repeated until 20–25 nucleotides are attached to each other. This approach is also known as the in situ synthesis method [1,48]. In the case of other biomolecule microarrays (protein and peptide microarrays), an active functional group is created followed by biomolecule immobilization.

**Figure 1:**
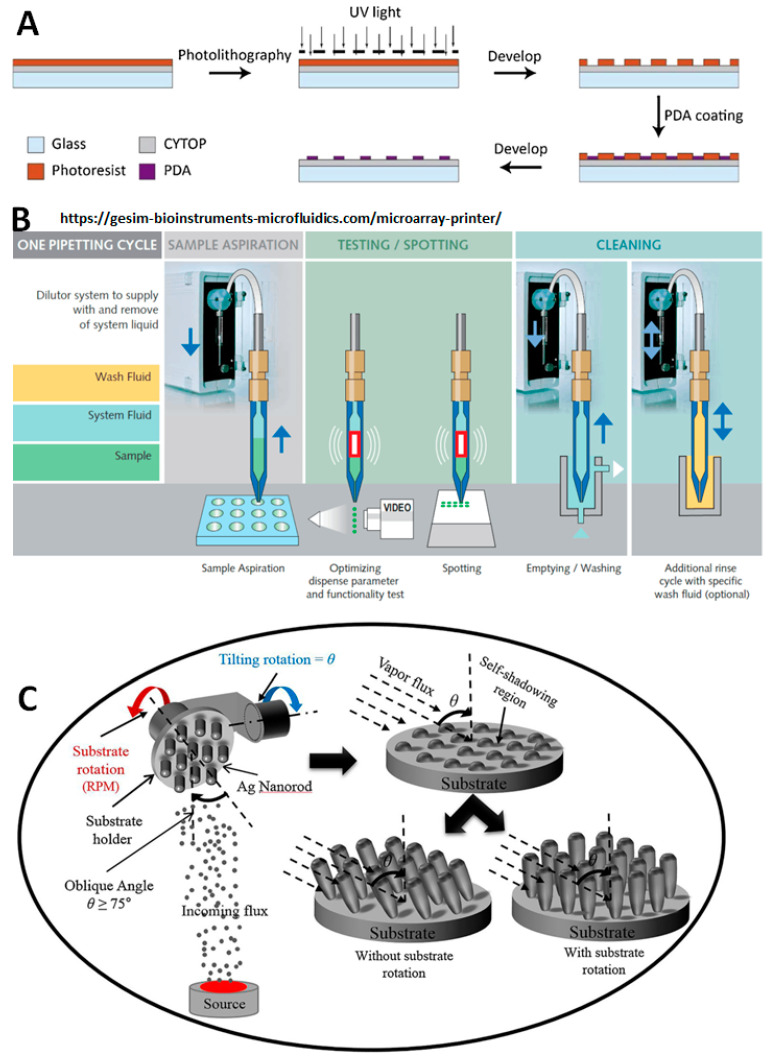
Different microarray printing strategies: (**A**) Fabrication of PDA patterned CYTOP (perfluoropolymer) glass slides via photolithography approach (reprinted with permission from [74]), (**B**) a representative of various steps involved in inkjet printing process (taken from https://gesim-bioinstruments-microfluidics.com/microarray-printer/; Accessed on 12 December 2022), and (**C**) GLAD method used to fabricate silver nanorods on glass slides (reprinted with permission from [93]).

High-density RNA microarrays are fabricated using the in situ synthesis method [49]. Antibody microarrays are fabricated on glass slides [50], where CYTOP (an amorphous fluoropolymer) is coated followed by spin coating a photoresist layer and applying a photomask. Subsequently, UV irradiation (365 nm wavelength), polydopamine (PDA) coating, and photomask removal result in a PDA patterned microarray chip (Figure 1A). Finally, antibodies are immobilized on the PDA patterns. Peptide microarrays [51] are fabricated in a Maskless Array synthesizer (Roche NimbleGen, USA) using the in situ photolithography approach.

***Mechanical microspotting*** is performed by releasing small volumes (µL, nL, or sub-nL) of the biomolecule-containing solution as spots on the desired area of the substrate via a sample-loaded pin in contact mode. With the advancement of robotic array spotting systems, several thousands of spots can be achieved simultaneously. Several DNA microarrays [52,53,54,55,56,57,58,59], glycan microarrays [60,61,62,63,64], peptide microarrays [65,66], and protein microarrays [36,67,68,69,70] are fabricated using commercially available instruments, e.g., Smart Arrayer, OmniGrid contact microarrayer, Gene chip, Bio odyssey, BioRobotics MicroGRID II microarray printer, etc.

In one study, a 200 nL solution comprising a mixture of different aptamers and antiaptamers was manually spotted on an epoxy-functionalized silicon chip to fabricate an aptamer microarray [71]. Similarly, in a few other studies, different glycans were manually spotted or printed using pins on epoxy glass surfaces [72], nitrocellulose-coated glass slides [73], poly-hydroxyethylmethacrylate (pHEMA)-cyanuric acid (CC)-modified glass slides [74], and N-hydroxysuccinimide (NHS)-activated PMMA-modified glass slides [75] to fabricate glycan microarrays. Additionally, DNA and glycoproteins were manually printed on an NHS activated PMMA-modified glass slide [75].

***Inkjet printing*** employs an automated printer fitted with piezoelectric dispenser tips loaded with the biomolecule solution. The use of an electric current dispenses a precise volume (µL, nL, or sub-nL) of spots on the substrate surface in noncontact mode. Similar to mechanical microspotting, when using inkjet printing, several thousands of spots (with different concentrations of biomolecules or different biomolecules) can be printed on a single chip [76,77,78,79]. DNA [46,77,80,81], antibodies [82,83], glycans [84], proteins [40,78,79,85], and peptides [86,87] are fabricated using SciFLEXARRAYER, Nano-Plotter, and Arrayjet. Figure 1B depicts a generic overview of the inkjet printing process. Clancy et al. [88] studied the quality of printed spots on epoxy-, amine-, octyl-, and fluorofunctionalized glass slides via inkjet, pin, and microcontact printing. Inkjet printing has produced quality protein spots (IgG and BSA, with varying sizes and ionic and hydrophobic properties) on all the surfaces as compared to other printing approaches. Additionally, printing on amine-functionalized glass using droplet-based techniques has produced superior quality spots than any other surface. Zhang et al. [89] fabricated a DNA microarray using the Personal Arrayer 16 microarray spotter (Biotools B&M Labs S.A, Madrid, Spain), where both contact printing and noncontact dispensing is available.

Researchers have also explored new strategies to fabricate microarray structures on glass and silicon surfaces. Chemical vapor deposition (CVD) [90,91] is an excellent thermal method to prepare 2D structures. Here, the coating material is heated above its melting point, and the generated vapors are directed onto the substrate surface for chemical reaction. This method does not require any expensive equipment. Petralia et al. [92] fabricated a microarray chip on a silicon wafer in a multistep process. The CVD method was used to create layers of alumina and SiO_2_ followed by sticking a polycarbonate ring and transparent adhesive tape. Sola et al. [77] introduced a bifunctional (NHS and azide) polymer (N,N′-dimethylacrylamide based) coating to silicon slide substrates to coimmobilize proteins and peptides to decrease the number of analyses, errors, and cost. Badshah et al. [93] implemented the glancing angle deposition (GLAD) method and physical vapor deposition method to fabricate highly porous silver nanorod arrays on glass. Silver deposition was performed by rotating the glass at an incident oblique angle of 75° (Figure 1C). Compared to glass substrates with slant NRs and commercially available amine-functionalized substrates, the fluorescence signal on this Ag nanorod array was enhanced drastically. Zhao et al. [94] performed a series of sequential photoinitiation steps to fabricate azide (N_3_)-functionalized spatially distributed hierarchical polymer microarray structures. These azide functional arrays can be used for biomolecule immobilization (discussed in Section 2.2).

In various studies, commercially available microarrays, such as the Explorer Antibody Microarray (ASB6000, Full Moon Biosystems, Sunnyvale, CA, USA) with 656 antibodies [95], VaxArray Coronavirus SeroAssay kit (#VXCV-5100, InDevR, Boulder, CO, USA) [96], Human Cytokine Antibody Microarray slides (AAH-CYT-G4000 kit, RayBiotech, Peachtree Corners, GA, USA) [97], Biotin-labeled Human antibody microarray (RayBio Biotech, Guangzhou, China) [98], Protein Microarray Slides (Grace Bio-Labs, Bend, OR, USA) [99], *HuProt* human proteome microarrays (CDI laboratories, Mayaguez, PR, USA) [100], SBC Mouse ceRNA microarray V1.0 (Shanghai Biotechnology Corporation, Shanghai, China) [101], and Agilent Whole Mouse 44 K ver. 2.0 microarray (Agilent technologies, Santa Clara, CA, USA) [102], were used.

In some studies, microarrays with active functional groups were purchased and used directly or after further modifications for biomolecule printing. The list includes CodeLink slides (SurModics, Eden Prairie, MN, USA) [76], Nexterion H slides (Schott, Mainz, Germany) [68,76], 3D-NHS slides (PolyAn, Berlin, Germany) [76], indium tin oxide slides [103], amine functional glass slides (Corning, Corning, NY, USA) [36], and ester functional plastic substrate (SBio^®^ PrimeSurface^®^, Tokyo, Japan) [104,105]. Marcelo et al. [106] have used the glycan array chip from the Consortium for Functional Glycomics (http://www.functionalglycomics.org; accessed on 20 November 2022).

### 2.2. Biomolecule Immobilization Strategies

**Scheme 1:**
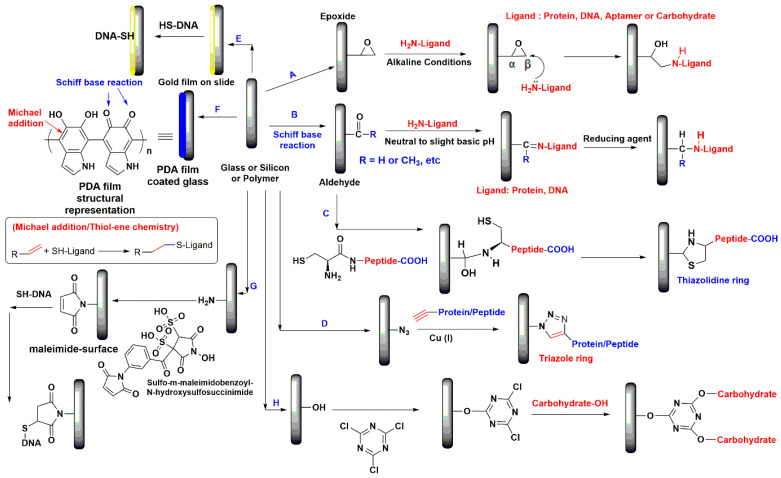
Overview of various chemical strategies used to modify the substrate surface and biomolecule (DNA, protein, antibody, glycan, and aptamer) immobilization. Regioselective ring opening chemistry (**A**); schiff base chemistry (**B**,**C**); click chemistry (**D**); sulfur-gold chemistry (**E**); thiol-ene chemistry (**F**); combination of NHS chemistry and thiol-ene chemistry (**G**); and nucleophilic substitution reaction (**H**).

The immobilization of DNA, antibodies, proteins, peptides, glycans, or aptamers on the microarray spot is the final and pivotal step in the microarray fabrication process. In order for the above-mentioned biomolecules to be immobilized, the surface of the microarray spot should possess readily available and active functional groups, such as epoxides, amines, azides, aldehydes, NHS, or thiols. The choice of functionalization depends on the chosen probe, sample requirements, working chemistry, and sensitivity requirements. The substrate materials often demand higher compatibility along with low nonspecific binding properties. In this section, we present a general schematic representation (Scheme 1) of the DNA, protein, peptide, antibody, glycan, and aptamer immobilization approaches adopted by researchers to develop the respective microarrays.

Under alkaline or acidic conditions and in presence of a nucleophile, epoxide-bearing molecules undergo a regioselective ring-opening reaction [40,41,62,63,66,78,79,92,107,108,109]. This chemistry is widely explored to functionalize porous materials, flat surfaces, etc., with various biomolecules [110]. Here, under alkaline conditions, a primary or secondary amine-functionalized ssDNA [59], aptamer [71], carbohydrate [72], or protein [67,111] reacts with the epoxide functional substrate in the least hindered position of the epoxide, i.e., the β position (Scheme 1A). This results in the covalent attachment of the biomolecule to the substrate.

Schiff base chemistry, a two-step process, is one of the oldest and most popular chemical methods to covalently attach the amine and carbonyl groups of aldehyde or ketone molecules to each other [89,112,113,114]. The first step involves the reaction of an aldehyde with an amine in neutral to slightly basic pH conditions. This results in the formation of an unstable intermediate imine (-C=N-). In the second step, the use of a reducing agent, such as sodium borohydride, converts the imine into an amine (Scheme 1B). In the case of microarrays, aldehyde-functionalized surfaces (glass and silicon) that are either commercially available or prepared in-house are used [69,70]. Amine-functionalized DNA [52,54,57,58,59] or antibodies with naturally existing amine groups [82] are immobilized in the above-mentioned two-step process to form DNA or antibody microarrays. Similarly, this chemistry can be further extended to a reaction between cysteines and aldehydes as well [115]. The final obtained molecule has a five-membered thiazolidine ring in its structure. Jian et al. [65] exploited this strategy and immobilized peptides containing a cysteine at the N-terminal point on aldehyde-functionalized glass slides (Scheme 1C).

The term click chemistry herein refers to “azide—alkyne cycloaddition” reactions. During the last two decades [77,116,117,118], it has been one of the most explored chemistries in various fields due to its simplicity, high product yields, and faster process to covalently link molecules. The chemistry involves the reaction of an azide (N_3_) molecule with an alkyne (-C≡C-) molecule in the presence of a copper (Cu (I)) catalyst. This leads to the formation of a molecule possessing a triazole ring in its structure (Scheme 1D). Zhao et al. [94] immobilized alkyne-functionalized peptides/proteins on azide-functionalized polymer-brush-coated silicon slides. Brambilla et al. [83] employed a DNA directed immobilization approach to convert a DNA microarray into an antibody microarray. In this approach, “antibody conjugated DNA” was prepared by conjugating azide-functionalized ssDNA with an alkyne-functionalized antibody. The obtained “antibody conjugated DNA” was hybridized with probe DNA functionalized silicon chips.

Thiol-ene [119,120] and sulfur–gold chemistry [121,122] are two chemistries widely exploited in the field of surface chemistry. Here, under optimized conditions, the molecules containing a thiol (-SH) in their structure readily react with dienes (-C=C-) or gold (Au) structures/surfaces. Simon et al. [53] grafted thiol-labeled DNA onto gold chips (Scheme 1E). Proteins/peptides can be immobilized on polydopamine (PDA)-coated films via Michael addition (thiol-ene) and Schiff base approaches [123,124]. Liu et al. [50] immobilized proteins/antibodies on PDA film-coated glass slides (Scheme 1F).

Rivas et al. [80] immobilized amino functional DNA covalently on the surface of a nitrocellulose membrane via UV irradiation. Wolf et al. [56] fabricated a DNA microarray on amine glass slides in a two-step process (Scheme 1G). The first step involved NHS chemistry [75,76,106,125], where a sulfo-m-maleimidobenzoyl-N-hydroxysulfosuccinimide ester reacted with an amine group to form a maleimide functional surface. The second step involved a thiol–maleimide reaction [126], where the thiol group of DNA reacted with a maleimide group present on the glass slide. Jing et al. [61] synthesized maleimide-functionalized carbohydrates and immobilized them on thiol-coated glass slides to fabricate a carbohydrate microarray.

CC is a heterocyclic triazine ring compound that contains three chlorine molecules in its structure. Liu et al. [74] prepared a glycan chip by using CC as a linker molecule to covalently (*via* a nucleophilic substitution reaction) attach carbohydrates to a hydroxyl methacrylate (pHEMA)-polymer-coated substrate (Scheme 1H). The advantage of this approach is the nonrequirement of carbohydrate (e.g., mannose, galactose, dextran, etc.) derivatization prior to its immobilization.

Streptavidin, a tetrameric protein and the homologous protein of avidin, is widely known for its remarkable ability to bind up to four molecules of biotin with a high affinity [19,127,128]. The interaction between streptavidin and biotin occurs via various noncovalent bonds, such as multiple hydrogen bonds and Van der Waals interactions, together with the ordering of surface polypeptide loops, which can bury the biotin in the interior of the protein [19,129,130]. This chemistry is used not only to immobilize the target biomolecule but also to conjugate fluorescence dye molecules. In the case of microarrays, several fluorescent dyes were conjugated with streptavidin (avidin-Cy3 [66,97], streptavidin-FITC [99], streptavidin–Alexa Fluor [68,98], streptavidin–fluorescent dye CF555 or CF647 [78,79], and streptavidin-Cy5 [100] conjugates) and are used to detect the biotin-labeled biomolecules present on the surface of a substrate.

Jeon et al. [99] used an aptamer microarray for the covalent and oriented immobilization of antibodies through the systematic modification of a photoactivatable Fc-binding RNA hybrid aptamer. In the work, the group developed various photoactivatable compounds (4-Maleimidobenzophenone, Maleimido-diazirine, Maleimido-5arm-diazirine, Maleimido-phenyldiazirine) and conjugated them onto thiol-modified Fc-binding antibodies.

### 2.3. Detection Methods

**Figure 2:**
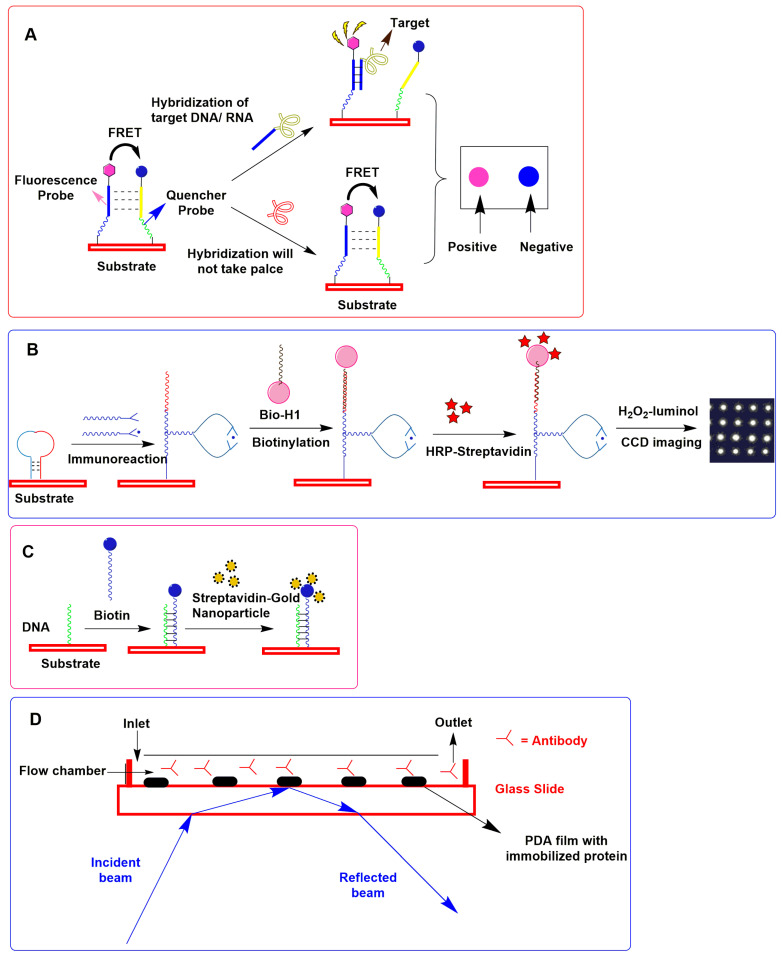
Detection methods: (**A**) FRET based approach to detect target DNA. In presence of target DNA only, the existing complex of fluorescence dye (Cy3) and quencher (BHQ2) is disrupted and generates fluorescence signal ([104]). (**B**) CL based enzymatic approach. This approach involves multiple steps: immunoreaction, HCR amplification, enzyme conjugation, and enzyme-CL (luminol p-iodophenol) substrate reaction ([52]). (**C**) Colorimetric detection (use of streptavidin-coated gold nanoparticles) of isothermal DNA amplification. Interaction of biotin and streptavidin was explored ([80]). (**D**) OIRD, a label-free detection method to study protein/antibody interactions with target molecules on a protein microarray chip ([67]).

Once the desired biomolecule microarray is fabricated and subjected to target capture, the next step in the process is their detection [131,132,133] and data analysis. In general, the detection methods are categorized as label-dependent and label-free. The use of fluorescent dyes (Cy3, Cy5, FITC, Alexa fluor 647, Alexa fluor 555, and Alexa fluor 488), radioisotopes (^32^P, ^33^P, and ^14^C), near-infrared dyes (phthalocyanine dyes, polymethine carbocyanine dyes, IRDye 800CW, IRDye 750, and IRDye 800), and enzymes (horseradish peroxidase) falls under the category of label-dependent. Importantly, fluorescent dyes (Cy3 label, Cy5,FITC, Alexa flour 647, Alexa fluor 555, Alexa 488, CF555 or CF647, DyLight^®^ 650, DyLight^®^ 550 [69], and Green 540 [95]) were preferred in most of the microarray articles [46,49,54,56,57,58,61,65,71,72,73,77,82,83,84,93,97,134,135,136]. The reason for this could be their ease of availability, the established protocol to conjugate the dyes, their use for designing a multiplex assay, and the commercially available screening instrumentation (confocal microscope, LuxScan 10K, and GenePix pro 6). Taguchi et al. [104] explored the concept of FRET (fluorescence resonance energy transfer) and designed signaling probe pairs (Cy3 based fluorescence and a BHQ2 based quencher) on a microarray chip to detect DNA and RNA without amplification. The hybridization of the target DNA/RNA fragment interrupted FRET and led to a fluorescence signal (Figure 2A).

Two studies related to DNA microarrays used an enzyme-based approach. In the first study [52], horseradish peroxidase catalyzed H_2_O_2_-luminol and produced a chemiluminescence (CL) signal that was recorded with a charge-coupled device. In the second study [55], streptavidin-conjugated HRP was bound to the array followed by substrate conversion and the detection of a change in color. In the case of an antibody microarray study [50], HRP labeled anti-IgG was used as a secondary antibody, and luminol was used as the chemiluminescent substrate. HRP oxidized luminol to 3-aminophthalate, which emitted light at a 425 nm wavelength (Figure 2B). In a study that dealt with DNA microarrays [80], streptavidin-coated gold nanoparticles were used as detection molecules (i.e., a colorimetric detection approach; Figure 2C). Here, a flatbed scanner (CanonScan 9000F Mark II, canon) was used to scan the microarrays. A similar instrument was used to screen the carbohydrate microarrays [137], where monoclonal antibodies and carbohydrate-binding modules were used as probe molecules.

SPR, RIfS, and OIRD are some of the label-free detection methods. The major advantage of these methods is their ability to acquire real-time biomolecular interactions and to perform high-throughput analyses. For example, SPR and RIfS measure thin film’s optical dielectric response resulting due to changes in the physical or chemical properties of a biomolecular interaction. OIRD is a highly sensitive detection system that measures the reflectivity difference between S- and P-polarization during a biomolecular interaction (Figure 2D). Zhong et al. [67] coated a PDA film on a glass surface with a thickness of 90.2 nm to enhance the sensitivity of a protein microarray during an OIRD analysis. The sensitivity of the array was enhanced by two orders of magnitude in comparison to the silane-coated glass slide. In the case of a DNA microarray [77], an LED based interferometric reflectance imaging sensor (IRIS) was used to study whether the immobilization of probes was successful or not. IRISs provide real-time information on the change in mass on the probing surface. During the surface plasmon resonance imagining of a DNA microarray [53], the use of a ruthenium (III) hexamine complex (RuHex) enhanced the overall refractive index. This is because RuHex, with its small size, accessed the inaccessible DNA layers within the microarray.

## 3. Application of Microarrays

In this section, we focus on the application of different biomolecule-based microarrays, i.e., nucleic acid microarrays, protein microarrays, peptide microarrays, glycan microarrays, antibody microarrays, and aptamer microarrays, since 2018 onwards.

### 3.1. Nucleic Acid Microarrays

Nucleic acids (DNA and RNA) are the most important biomolecules found in all living cells and viruses with the primary function of storage and carrying genetic information [138,139]. Their chemical structure comprises of heterocyclic aromatic bases conjugated to a sugar–phosphate backbone, which is involved in a phosphodiester bond. Nucleic acid arrays, also known as DNA arrays, involve specific sequences of “probe” DNA molecules that are synthesized or deposited onto a solid flat surface [20,21,22]. The application of these microarrays is to identify the target DNA sequence and to measure its concentration in a solution. In recent years, the applications of DNA have widened, allowing for gene expression analyses, transcription factor binding analyses, and genotyping. More specific details about DNA microarray fabrication can be referred to [23,45,48,140]. The articles in this section are reviewed based on their purpose of analysis, such as eukaryote and prokaryote, respiratory disease, and biomarker identification; the identification of gene mismatches and viruses; species authentication; and developing new strategies for DNA immobilization.

An 18S rRNA phylogenetic microarray [135] detected eukaryotic organisms from marine sediments by targeting 18S rRNA operational taxonomical units (OTUs). The limitation of this approach was a decrease in the specificity with increasing sequence similarity. Using a DNA vertical flow paper microarray [80], *neisseria meningitidis* was detected with copies (38 and 2 × 10^6^ per vertical flow assay) that were significantly similar to the Loop-Mediated Isothermal Amplification assay [141]. *Salmonella enterica* was detected even at low levels (<10 colony forming units (CFUs)) in leafy greens using the DNA microarray-based PathogenDx system [142]. A FRET based DNA microarray system [104] specifically detected both 16S rDNA and 16S rRNA from *E. coli* in 60 min.

The potential of the automated electronic microarray platform [46] was explored to detect and differentiate multiple pathogens in a bovine respiratory disease complex and bovine enteric disease in a single sample. The microarray platform displayed a level of detection and differentiation of multiple pathogens similar to the multiplex PCR/RT-PCR approach. Six swine pathogens (PRDC, PCV2, PRRSV, Mhp, APP, HPS, CSFV, PPV, and SIV) were also simultaneously detected using an oligonucleotide microarray [57].

**Figure 3:**
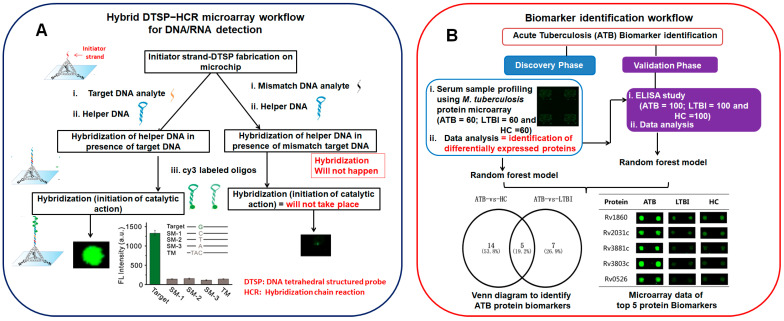
(**A**) A pictorial workflow presentation of hybrid tetrahedral DNA structured probe in conjugation with hybridization chain reaction (DTSP-HCR) concept used to distinguish single-base mismatches in DNA ([89]) and (**B**) an overview of acute tuberculosis (ATB) biomarker identification using a two-phase strategy (discovery phase and validation phase) ([149]). Venn diagram and microarray chip visual analysis revealed the potential of 5 protein biomarkers to distinguish ATB and LTBI/HC. LTBI represents latent tuberculosis infection, and HC represents healthy control.

A DNA microarray based on hairpin DNA molecules [52] detected three protein biomarkers (carcinoembryonic antigen (CEA), α-fetoprotein (AFP), and carcinoma antigen 125 (CA125)) even at a low concentrations. These detection levels were either similar [143,144] or significantly higher [145,146] than those of earlier reported studies. Hypothermia-exposed murine lung samples were analyzed using the Agilent technologies DNA microarray platform (mouse, human, cDNA, oligo, number of genes) to identify the forensic biomarkers [102]. The outcome of this study was the identification of 4094 genes (1699 upregulated and 2395 downregulated genes) that exhibited hypothermia-induced differential expression in the lungs. A circular RNA microarray-based [101] functional experiment revealed cicRNA 7079 as a new antiapoptotic molecule in traumatic spinal cord injuries in mice. The differential DNA methylation profiles [136] in Wharton’s jelly mesenchymal stem cells (WJ-MSCs), 5′Azacytidine treated WJ-MSC, and human cardiac tissue were studied using a customized 180 K human DNA methylation microarray. Catalytic hairpin assembly involves the use of a hairpin-shaped DNA, which, upon interaction with the target single-stranded analyte, leads to fluorescence signal amplification. An ultrasensitive fluorescence DNA microarray platform [89] mediated by a tetrahedral DNA structured probe in combination with a hybridization chain reaction (HCR) was developed to detect both DNA and miRNA. The LOD of this method is 10 aM, and it distinguishes even a single-base mismatch in DNA (Figure 3A). This approach [58] was applied to a microarray platform to simultaneously detect multiple miRNAs in spiked human serum and the pathological cells Hela (cervical cancer cells) and MCF-7 (breast cancer cells) within 60 min. A silicon-based microarray chip (Si@Al@SiO_2_ layered with a probe density of 1.9 ± 200 molecules μm^−2^) [92] was fabricated and validated by detecting melanocortin 1 receptor single-nucleotide polymorphisms.

A *BRCA1* gene DNA probe microarray containing silicon nanowires [59] successfully discriminated the model mismatches in the sequence of *BRCA1*. The synthetic DNA of seven antibiotic resistance genes from five cell lysates and three *E. coli* strains were detected using an oligonucleotide microarray [56]. The simultaneous detection of six avian influenza virus genes was achieved using an oligonucleotide microarray [81]. A DNA microarray [147] identified the serotypes of 14 *E. coli* isolates, and 6 *E. coli* isolates were found to be Shinga toxin-producing *E. coli*. Of the 36 identified virulence genes, *hemL*, *lpfA*, and *iss* were found to be more prevalent.

Hepatitis B virus (HBV) DNA was detected using the hybridization-induced silver nanoparticle (AgNP) aggregation strategy [54]. This strategy involves the use of AgNPs conjugated with thiol-functionalized cy3-probe and hybrid nucleic acid probes (Tag-A and Tag-B). The presence of HBV DNA on the microarray only leads to AgNP aggregation and amplifies the fluorescence signal. In this case, a 1560-fold signal enhancement was achieved.

The ArrayTube2 DNA microarray [55] with *cytb* and 16S rDNA probes authenticated 10 different food fish specimens from a total of 67 fish species within 5 h. An oligonucleotide microarray [148] was used to authenticate five marine mammal species (dolphins, seals, sea lions, white whales, and finless porpoises) in food and feed.

A multipolymer microarray spot platform [77] with 16 different copolymers and various functional groups (amines, azides, or dibenzocyclooctynes) was explored for DNA probe immobilization. This DNA microarray platform resulted in improving the microarray sensitivity to be greater than that of the other existing immobilization approaches.

### 3.2. Protein Microarrays

Proteins are a class of biological macromolecules comprising of hundreds or thousands of amino acids covalently linked to each other via peptide bonds [150]. Each protein is unique, with its own three-dimensional structure and amino acid sequence. In our body, they perform various useful functions (store and transport molecules, act as catalysts, transmit signals, provide strength to muscles and tissues, and protect us from infections). Proteins are immobilized on microarrays (protein microarrays) and explored to unravel proteome complexity and to identify protein functions [26,133,151,152]. They are classified into analytical, functional, and reverse-phase protein microarrays. However, an analytical protein microarray involves the use of antibodies. Functional protein microarrays are applied to study protein–protein interactions, enzyme–substrate relationships, biochemical activity, and immune responses. The fabrication of these microarrays is challenging due the requirement of a high amount of purified proteins. Hence, proteins are expressed in yeast and *E. coli* expression systems. Reverse-phase protein microarrays (RPPAs) are high-throughput platforms that comprise of target proteins from cell or tissue lysates, sera/body fluids, or subcellular fractions immobilized as spots on the substrate [153,154,155]. The subsequent step involves the use of specific tagged antibodies to detect the posttranslational modifications of proteins (e.g., phosphorylation) or the total protein expression levels. Considering the availability of the amine and carboxyl groups within the protein structure, the immobilization of proteins on a surface is relatively straight forward. Readers are referred to Section 2.2 for protein immobilization chemistries. The articles in this section are presented based on their purpose of analysis, such as biomarker identification, mechanistic insights on infections, the study of antifouling properties, glycosylation profiling, the study of drug-binding proteins, vaccine candidate identification, target molecule detection, and the detection of SARS-CoV2 antigens in patient sera.

RPPAs [36] were used to quantitatively screen protein biomarkers from hepatocellular carcinoma (HCC) patients’ sera. From the analysis, it was concluded that six proteins were identified with the potential to become diagnostic markers for HCC. A tuberculosis protein microarray [149] was explored to screen the serum samples of active tuberculosis (ATB) patients, latent tuberculosis infection (LTBI) patients, and healthy controls (HCs). Four potential protein biomarkers were identified that can distinguish between ATB and LTBI, with a 93.3% sensitivity and 97.7% specificity (Figure 3B).

The biological mechanism of the yang-deficiency constitution (intolerance to cold) at the protein level was studied using a protein microarray [98]. The outcome was the identification of 85 differentially expressed plasma proteins (64 upregulated and 21 downregulated proteins), and it indicated that (low immune) metabolic disorders and endocrine disorders are the potential reasons. A protein microarray [68] was evaluated to understand if it could provide more insights into pigs’ serological responses to *Mycoplasma hyopneumoniae* infections. Compared to the commercial ELISA, the protein microarray proved to be an alternative and sensitive tool to detect *M. hyopneumoniae* infections.

Diverse biomolecules (fibronectin, BSA, streptavidin, and RGD peptides) were immobilized on a functional hyperbranched PEG (polyethylene glycol) layer on silicon slides [94] to achieve the antifouling property. BMSC and mouse L929 cells were successfully cultured on the microarray.

A lectin-based protein microarray [78] was explored to obtain the glycosylation profile of the insulin-like growth factor receptors (IGF1 and IGF2) in colorectal carcinoma tissues. One of the important observations was that both receptors possessed high levels of α2,3 sialic acid residues; low levels of tri-/tetra-antennary complex type N-glycans with Gal β1,4 GlcNAc β1,6 Man; and high-mannose structures with terminal mannose. Similarly, a lectin-based protein microarray [79] was used to analyze the glycosylation of recombinant dimeric IgA1 antibodies under the influence of different media supplements (asparagine, glutamine, and succinic acid). The absence of sugars (D-(+)-glucose, D-(+)-galactose, and D-(+)-mannose), use of one supplement, or replacement of one supplement with another resulted in glycan level fluctuations or changes in the glycan forms.

A *HuProt* human protein microarray [100], in combination with a bioinformatics analysis, was used to screen and identify potential doxorubicin (Dox; an anti-tumor drug)-binding protein targets. A total of 27 proteins were shortlisted, and studies on one of the proteins, HRAS (Harvey rat sarcoma viral oncogene homolog), revealed that Dox promotes HRAS-RAF complex formation.

A *Neisseria meningitidis* antigen microarray [85] was used in human phase I clinical trials to identify candidate vaccine proteins.

A macroporous polymer with a hydrophilic–hydrophobic property served as a layer for preparing protein arrays [111]. Increased polymer layer hydrophilicity increased the analytical performance of the protein array. An acetylcholinesterase (AChE) antibody-coated chip was successfully applied for both AChE detection and the study of various enzyme kinetic assay parameters.

A SARS-CoV-2 variant protein microarray [70] was used to profile the humoral immunity in a vaccinated (unvaccinated, partially vaccinated, and fully vaccinated) population. The major outcome was that full vaccination provided surrogate neutralization against all the mutants. The behavior of the immunoglobulins (IgG, IgA, and IgM isotypes) varied throughout the vaccination process. A coronavirus antigen microarray (COVAM) was fabricated using various recombinant proteins and antibodies [156]. The COVAM selectively discriminated the viral (SARS-CoV-2 and influenza) infections with similar symptoms and showed a 77.2% sensitivity and 100% specificity. A flow-based chemiluminescence microarray immunoassay chip was developed using existing knowledge [157,158] for the identification of the SARS-CoV-2 IgG antibodies in human serum and plasma [159]. Compared to the recomLine and recomWell systems, this microarray platform showed a 100% specificity and sensitivity within 8 min. In a protocol development study, a SARS-CoV-2 antigen-fabricated microarray was used to study the feasibility of assessing the interaction between the antigens and various immunoglobulins (IgG, IgM, and IgA) in patient sera [160]. Another protocol study discussed the use of a SARS-CoV-2 antigen-fabricated microarray [161] to profile protein sera samples. The study involved the preparation of proteins, microarray fabrication, sera profiling, and data analysis.

### 3.3. Peptide Microarrays

A peptide is a series of 2–50 amino acids covalently linked to each other via a dehydration reaction between the amino (-NH_2_) moiety of amino acid A and the acid (-COOH) moiety of amino acid B. Peptides offer several advantages (ease of preparation, chemical stability, and compatibility with various immobilization strategies) over proteins [32,162]. Synthetic peptides are considered to be protein mimics and can be used for different biomedical applications [163,164,165,166]. Peptide-based array systems were first reported in the early 1990s [12,167], and, over the years, they have been used for studying enzyme functionality and inhibitor screening, the identification of disease biomarkers, and drug development. Peptide microarray synthesis can be performed in two ways [32,168]. The first approach is to perform in situ stepwise peptide synthesis on the array itself. The second approach is to spot or print the presynthesized peptides on the array slides via contact or noncontact mode. Here, the synthesis of peptides is performed via the widely known Merrifield solid-phase peptide synthesis technique. The second approach is mostly preferred over the first approach because its presynthesized peptide purity is excellent. The trend of preparing a peptide microarray via the second approach has persisted even in the articles published since 2018 [51,65,66,86,87,169,170]. Readers are referred to Section 2.2 for detailed peptide immobilization strategies. The articles in this section are presented based on their purpose of analysis, such as the identification of allergen-specific peptides, biomarker quantification, extracellular vesicle phenotype characterization, and the profiling of the SARS-CoV-2 antigen.

An allergome-wide 16-mer peptide microarray was constructed to detect allergen peptide-specific IgE, IgG4, and IgG [51]. The outcome was the identification of allergen-specific humoral immunity.

A peptide microarray on a glass slide was designed to quantitatively screen the matrix metalloproteinase-2 secretion levels in normal cells (human colon epithelial cells) and four cancer cells (cervical, colorectal, hepatoma, and osteosarcoma cells) [65]. The obtained microarray sensitivity was superior to the existing commercial kit. This was further improved by immobilizing peptides on a zinc oxide nanorod-polymer brush composite-grafted substrate [66]. An antibody and peptide co-immobilized microarray was developed to study HEK derived extracellular vesicle (EV, membrane-bound vesicles) phenotype characterization [87]. Compared to immobilized antibodies, the bradykinin-derived peptide displayed high binding to EVs.

A SARS-CoV-2 peptide microarray with peptides that were 15 amino acids long with a 5-amino-acid overlap was developed [169]. The approach of peptide synthesis involved two steps, where the reference sequence of the SARS-CoV-2 genome, encoding 10 proteins, was identified and where a peptide library was prepared. The microarray was successfully applied for the epitope mapping of the IgM and IgG antibodies in COVID-19 infected patients’ sera. One of the identified peptide epitopes (FRKSN) could help in neutralizing RBD-ACE2 interacting antibodies. A peptide microarray against COVID-19 patients’ sera was constructed via the phage immunoprecipitation sequence (screened COVID-19 patients’ sera with nine human coronavirus genomes), ReScan (scanned patient serum antibodies and produced phage-displaying immunogenic antigens, i.e., peptides), paper microarray fabrication, and the identification of nine peptide candidates [170]. The peptide microarray showed an 88% positive COVID-19 rate and a 75 and 100% sensitivity towards the sera of low- and high-neutralizing titers. A peptide sequence that recognizes the SARS-CoV-2 spike protein was designed, synthesized, and fabricated as a peptide microarray [86]. This microarray was applied to the profiling of the linear epitopes of the spike protein in COVID-19 patients’ sera. A spike variant protein microarray was developed for the screening of drugs, neutralizing activity, and profiling humoral immunity using the sera of COVID-19 patients with varying levels of severity [171]. Compared to severe patients, angiotensin-converting enzyme 2 (ACE2) inhibition was not strong in moderate or critical patients. The two ACE2 inhibitors (ramipril and perindopril) displayed dose-dependent inhibition in the case of all the spike variants, with the exception of B.1.617.3.

### 3.4. Glycan Microarrays

Glycans are a diverse chain of chemically linked monosaccharides present on the cell surfaces of all living organisms. These glycans are in conjugation with proteins (glycoproteins and proteoglycans) and lipids (glycosphingolipids). The interaction of glycans with glycan-binding proteins (GBP) helps in understanding the molecular mechanisms of various immunological events [172,173,174]. The first reported glycan microarray in the year 2002 [175,176] studied GBP events using a small amount of a glycan sample [28,29,177]. Later years witnessed the use of glycan microarrays in assessing glycan-processing enzyme characterization, the discovery of functional glycans, drug discovery, studying weak carbohydrate interactions, and pathogen diagnosis [76,178,179,180]. The immobilization of glycans on a solid surface is generally performed after the target glycan is modified with a reactive functional group (NHS, amines, alkenes, carbonyls, and thiols). The articles presented in this section are based on their purpose of analysis, such as glycan quantification; carbohydrate–protein (immunoglobulins, lectins, and the spike protein of SARS-CoV-2) interaction studies; the identification of viruses, foodborne bacteria, and enzyme activity; and IC50 value estimation.

Different strategies for glycan immobilization on amine functional silicon oxide and glass surfaces were studied [72], and the glycan quantity was quantified using a model lectin Concanvalin A. The glycan chip has a shelf-life activity of more than 5 months.

A glycan microarray [106] (from the Consortium for Functional Glycomics with 610 natural and synthetic glycans) was used to identify the secondary-binding site in human macrophage galactose-type lectin. Two different oligosaccharides ((**1**) Glc-(α-1,2)-Glc and (**2**) Man-(α-1,2)-Man-(α-1,2)-Man: central mannose with β-1,4) fabricated on three commercially available slides were used to study the weak-affinity interactions between carbohydrates and lectins [76]. Glycan microarrays with N-glycolylneuraminic acid (Neu5Gc) and N-Acetylneuraminic acid (Neu5Ac) glycans revealed that different intravenous immunoglobulin (IVIG) preparations show that IgA has cross-reactivity against several Neu5Ac-glycans and that anti-Neu5Gc has high specific IgG reactivity [63]. Carbohydrate microarrays with varying densities (mono-, bi-, and tetravalent dendrons) were fabricated via a spot-wise strain-promoted azide–alkyne cycloaddition approach and were evaluated for carbohydrate–lectin interactions [103]. With the increasing valency of the dendron structure from mono- to tetravalent, the binding affinity of the lectins (Pisum sativum, Wisteria floribunda, the extracellular domains of the human lectin receptors DC-SIGN, DC-SIGNR, Langerin, and Dectin-2) also increased. A carbohydrate microarray fabricated on a polymethylacrylic acid glass substrate [75] was studied for its affinity to the SARS-CoV-2 spike protein. The two carbohydrates heparin and fucoidan specifically recognized the spike protein of SARS-CoV-2. A glycan microarray consisting of 800 components identified 26 mAbs from a pool of 516 human mAbs [64]. The further analysis of these 26 mAbs provided insights on their cellular origins and binding specificities. This could help in designing future diagnostic kits and in the therapeutic application of various diseases.

A carbohydrate microarray [74] fabricated on CC-polyHEMA-slides had a high carbohydrate-loading ability and a low LOD of 9.28–928 µM as compared to the existing arrays. The two carbohydrates sialic acid and ManA2 interacted with the influenza A virus H1N1-hemagglutinin (an IAV envelope glycoprotein).

Various mannose-coated microarrays were constructed [61] to identify foodborne bacteria. A mannose array chip prepared on a thiol-functionalized glass displayed a better detection performance of the target bacteria over NHS functionalized glass slides. The mannose array chip showed the successful positive binding of 8 out of 12 *salmonella* isolates and 7 out of 9 diarrheagenic *E. coli* isolates with varying binding affinities. A total of 31 neutral N-glycans from ovalbumin were isolated and immobilized on a nitrocellulose-coated glass slide to develop a glycan array chip [73]. A glycan array chip with 6 *Helicobacter pylori* (*H. pylori*) lipopolysaccharides (LPSs) and 26,695 strains was constructed on a nitrocellulose-coated glass slide [84]. This glycan chip successfully detected the specific anti-*H. pylori* LPS IgG response and distinguished it from noninfected human sera. Hybrid-type N-glycans with bisecting GlcNAc showed a specific affinity to wheat-germ agglutinin, and complex-type N-glycans with bisecting GlcNAc displayed a low binding affinity to WGA.

**Figure 4:**
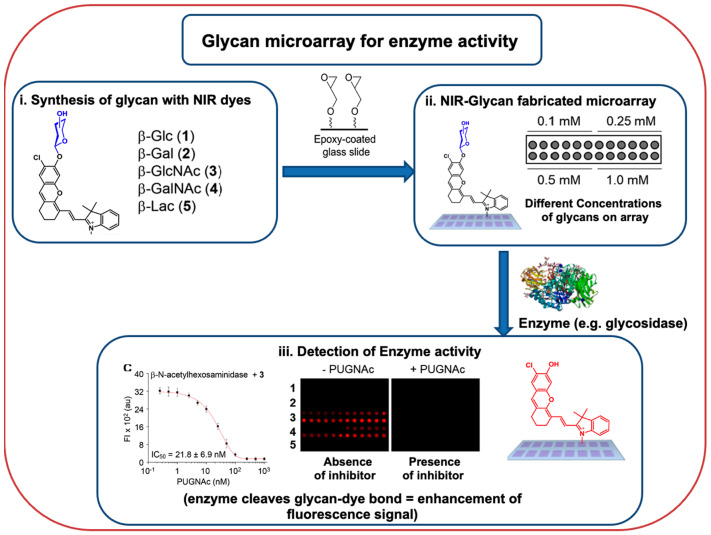
A generic workflow of the application of glycan microarray chip to efficiently profile enzyme activities and determine enzyme inhibitor IC_50_ values ([62]).

Glycosidase enzyme (β-glucosidase from almonds, β-galactosidase from A. oryzae, and β-N-acetylhexosaminidase from S. pneumoniae) activities were studied using a carbohydrate microarray (Figure 4) comprised of a series of different glycosylated near-infrared probes (five probes and five carbohydrates) [62]. These arrays were also explored to estimate the IC_50_ values of glycosidase enzyme inhibitors (CBE, DGJ, and PUGNAc).

### 3.5. Antibody Microarrays

Since the COVID-19 pandemic started, the word “antibodies” has become a much more familiar name in every household around the globe. Antibodies are Y-shaped protein molecules with a specific binding site for antigens. They are produced through hybridoma technology, which was introduced in 1975 by Kohler and Milstein [181]. This has resulted in the production of several monoclonal antibodies for therapeutic and diagnostic applications [182,183,184,185,186]. Antibodies and their derivatives (single-chain variable fragments, antigen-binding fragments, and nanobodies) are printed on microarrays to study the signaling pathways of various disease states, protein–protein interactions, and drug mechanisms and to identify the protein markers of autoimmune diseases, infectious diseases, and neurodegenerative diseases [34]. The performance of these antibody microarrays is significantly comparable to the conventional ELISA and Western blotting methods in terms of high throughput, multiplexing, sensitivity, and sample diversity. The articles presented in this section are based on their purpose of analysis, such as polysaccharide profiling, biomarker identification, target protein (interleukins (ILs), TNF-α, andSARS-CoV-2 antigens) and extracellular vesicle capture, and virus screening.

The comprehensive microarray polymer profiling approach uses a series of carbohydrate-binding modules and monoclonal antibodies to detect glycan epitopes. This strategy [60] is used to track polysaccharides during the winemaking process.

A novel biomarker CCL5 (out of 274 detected human proteins) from primary colorectal cancer tissue was identified using an antibody microarray [97]. Coronary artery stenosis serum protein profiling was studied using an antibody microarray [95] to identify the novel biomarkers associated with the disease. Irrespective of gender, the levels of cadherin-P, IL-5, glutathione S-transferase Mu, and neuronal nitric oxide synthase increased significantly. Additionally, the levels of nine proteins increased 4–30 fold, specifically in men only. The protein list included fibroblast growth factor 1, collagen alpha-1(II) chain, granulocyte–macrophage colony-stimulating factor, IL-1α, angiopoietin-2, granulocyte colony-stimulating factor, lymphocyte cell-specific protein tyrosine kinase, and kappaB kinase b. Similarly, in the case of women only, the levels of eight proteins decreased 4–15 fold. The protein list included cyclin-dependent kinase 1, DNA fragmentation factor subunit alpha, early E1A protein, calponin, ADP-ribosylation factor 6, alpha skeletal muscle actin, thyroid hormone receptor alpha, and alpha-methylacyl-CoA racemase. Using the *E. coli* proteome chip microarray [69], IgM and IgG antibodies were screened from 80 schizophrenia patient samples and 40 healthy individuals. They identified three potential antibody biomarkers (yjjU, livG, and ftsE) that could help in differentiating adult-onset schizophrenia and healthy individuals.

PDA microarray spots on a layer of amorphous fluoropolymer-CYTOP-glass slides [50] were used for protein (IgG as model molecule) immobilization and binding studies. Under optimized binding conditions, an anti-IL-6 protein microarray showed specificity to IL-6 in the presence of spiked IL-10. Interestingly, in one study, the explored aptamers were used [99] to immobilize antibodies and to create antibody microarrays. The applicability of this chip was demonstrated by immobilizing adalimumab, a TNF-α binding antibody. The antibody specifically bound to TNF-α and not to other proteins (IL-5, CD2, PD-L1, and TGF-*β*3). In comparison to conventional antibody microarrays, the antibody microarray developed via the DDI approach [83] efficiently captured the extracellular vesicles of the HEK-293 cells from the diluted solutions.

Six upper respiratory tract viral pathogens (IAV, IBV, RSV, hAdV, hPIV2, and hPIV3) were simultaneously screened using an antibody microarray [82]. The advantage of this microarray over the ELISA is the requirement of low reagent volumes and the multiplexing of six monoclonal antibodies on a single chip.

A MosaiQ COVID-19 antibody microarray [134] was used to detect antibodies against the SARS-CoV2 antigens in French patients’ samples (whole blood, plasma, or serum specimens). The overall sensitivity (88%) of the MosaiQ test was significantly higher than that of the other existing commercial competitors (< 79%). This microarray proved to be a high-throughput assay with a very high sensitivity and specificity, greater than those of the ELISA. The VaxArray Coronavirus SeroAssay kit, containing nine coronavirus spike proteins [96], was used to perform the simultaneous analysis of the antibody responses to all of the nine printed antigens in 2 h. This platform displayed a 0.32–1.99 ng/mL limit of quantification and a 76–911-fold linear dynamic range. A disposable “pre-equilibrium digital enzyme linked immunoassay (PEDELISA)” microarray platform was developed to monitor various cytokine (IL-6, TNF-α, IL-1β, and IL-10) levels in severely ill COVID-19 patients within 4 h [187]. The advantage of the PEDELISA over the other existing systems is its high sensitivity and interassay repeatability. Both the IL-6 and IL-10 levels in the patients receiving tocilizumab (IL-6 inhibitor) were heterogeneous and indicated the need for a personalized strategy.

A proteomic antibody microarray [188] was used to investigate the differentially expressed proteins (DEPs) in the human sera of people administered with three different traditional Chinese medicinal constitutions. For the phlegm-dampness constitution, the proteins were upregulated, and, in case of the phlegm-damp-heat constitution, the proteins were downregulated. This could be due to hyperthyroidism or a low immunity.

### 3.6. Aptamer Microarrays

Aptamers are small single-stranded DNA or RNA oligonucleotides that possess the special ability, similar to antibodies, of binding to the target molecules [189]. Since they are synthesized from nucleic acids and have an affinity similar to antibodies, they are known as chemical antibodies as well. The typical length of an aptamer is in the range of 20–60 or 80 nucleotides. The high affinities and specificities displayed by these aptamers to the target molecules, either a complex large protein or a simple small inorganic molecule, is similar to that of antibodies [190]. Hence, they are considered to be an efficient alternative to antibodies. Additionally, compared to antibodies, aptamer production is simple and cost-effective and has a high batch-to-batch reproducibility and stability and a very low immunogenicity [191,192]. Due to this added feature of having a very low immunogenicity, they are also considered to be potential agents for therapeutic and diagnostic applications [192,193]. Researchers also explore them in microarrays and study them with protein quantification [193]. Aptamers are synthesized through a process called the systematic evolution of ligands through exponential enrichment. For detailed information, readers are referred to [194,195,196]. The fabrication of aptamer microarrays is performed via two methods (the in situ synthesis method and the postsynthesis method). The in situ synthesis method involves the direct synthesis of aptamers on the microarray surface. In case of the postsynthesis method, the synthesized aptamers are spotted on functionalized (carboxyl, hydroxyl, and amine) surfaces via noncontact or contact mode. Considering that aptamers are alternatives to antibodies, it is interesting to note that they were also used as an alternative linker molecule to immobilize the antibodies on a microarray platform [99].

Microfungi are known to produce toxic secondary metabolites, known as mycotoxins, which are harmful to both humans and animals [197,198]. There are around 300 mycotoxins of which 6 (aflatoxins, trichothecenes, zearalenone, fumonisins, ochratoxins, and patulin) are majorly found in various foods, such as maize, wheat, rice, cereals, grapes, apples, etc. According to the guidelines from various public health and government bodies in the USA and the European Union, the allowed level of some of these toxins is as follows: aflatoxins (B1, B2, G1, and G2) are allowed at 20 µg/Kg in the USA and at 4–15 µg/Kg in total (2–12 µg/Kg for B1 alone), ochratoxin A is allowed at 2–10 µg/Kg in the EU (not set in the USA), fumonisins (B1, B2, and B3) are allowed at 200–1000 µg/Kg in the EU and at 2000–4000 µg/Kg in the USA, patulin is allowed at 50 µg/Kg in the EU and at 10–50 µg/Kg in the USA, and zearalenone is allowed at 20–100 µg/Kg in the EU (not set in the USA). Multiple mycotoxins (ochratoxin A, aflatoxin B1, and fumonisin B1) in spiked wheat, corn, and rice samples were simultaneously detected using the FRET approach [71]. With varying mycotoxins, the LOD of this system ranged from 0.21 to 15.4 pg/mL.

Organophosphorus pesticides are mainly used to protect crops from pests [199]. Over the years, their extensive use has caused more harm to human health [200]. According to the EU, the established maximum residue values of two of the pesticides, namely, phoxim and parathion, and isocarbophos in fresh vegetables and fruits are 50 µg/kg, 10 µg/kg, and 50 µg/kg, respectively [201]. A high-throughput fluorescence-based aptamer microarray was developed for the detection of multiple organophosphorus pesticides in food [202]. Thioflavin T was used as the fluorescence indicator, which was bound to the aptamer. The interaction of the target organophosphorus pesticides (phoxim, parathion, fensulfothion, and isocarbophos) with the aptamer resulted in the displacement of Thioflavin T and a decrease in the fluorescence intensity. The LOD of this microarray was 25.4 ng/mL for phoxim, 12.0 ng/mL for parathion, 7.7 ng/mL for fensulfothion, and 9.9 ng/mL for isocarbophos, respectively.

## 4. Conclusions—Future Directions

The research articles reviewed here (since 2018) indicate the broad use of five biomolecule (DNA, protein, glycan, peptide, antibody, and aptamer)-based microarray platforms in various studies. In the majority of the studies, only the existing fabrication methods were used. We think the reason for this was the good establishment (reliable literature data) of the existing methods, the wide commercial availability of the instruments, the digital automation of the various processing steps, and more interest in exploring the applications of the established microarrays. Two fabrication strategies, namely, the GLAD method and the sequential photoinitiation steps, were explored to fabricate microarrays. GLAD offers the fabrication of nanoarrays with a controlled shape, size, and porosity and with a slight modification of the incident angle, rate of evaporation, and time. The sequential photoinitiation step process has the advantage of printing 2D or 3D structures in a controllable manner and enhancing the available biomolecule immobilization reactive sites, thereby enhancing the sensitivity of the array and enhancing cell adhesion on the surface for the improved analysis of cellular responses. In the case of biomolecule (DNA, protein, peptide, glycan, antibody, and aptamer) immobilization, the existing approaches, such as epoxy, Schiff base, click, thiol-gold, and thiol-ene, were used. This is because of their wide success in previous studies and also the commercial availability of reactive-functional-group-coated slides. An interesting approach was the use of cyanuric acid, a low-toxic molecule, as a branching linker molecule. This approach enhanced the carbohydrate load on the substrate. High biomolecule loading enhances the analyte-binding efficiency and the improvement in array sensitivity. Streptavidin–biotin chemistry was explored for detection purposes (i.e., the use of streptavidin-conjugated dye to detect biotin-conjugated biomolecules). Fluorescence tags and enzyme-based methods are still the popular choice for detection studies. This is because of the wide number of commercial tags, it being a gold standard method, the availability of instrumentation, and its simplicity in performing the detection studies. Considering the advantages (the study of molecular interactions and time-lapse measurements) of the FRET principle, few studies have also exploited it. The obtained data is highly interesting and displays the potential of the FRET system. Although the popularity of using label-based detection approaches remained untouched, the potential of label-free detection methods (surface plasmon resonance imaging (SPRi) and OIRD) was also explored with good success. SPRi overcomes the limitation of SPR, can perform simultaneous studies of multiple interactions, and provides real-time data (binding kinetics), which is a limitation of fluorescence studies. Similarly, OIRD also offers real-time and high-throughput analyses with the added value of being nondestructive. The use of a PDA layer on the glass surface enhanced the sensitivity of OIRD to the nanogram levels.

Of the various DNA microarray methods developed, three methods, namely, the vertical flow paper method, the tetrahedral DNA structured probe in combination with a hybridization chain reaction, and the catalytic hair pin using FRET system, seem efficient alternative to the existing methods (Loop-Mediated Isothermal Amplification, a carbon nanoelectrode, and a chemiluminescence immunodevice). With respect to the microarray sensitivity, the tetrahedral DNA structured probe showed an excellent LOD (aM range), identifying even a single mismatch in DNA. Six different pathogens were simultaneously detected with a good LOD (10^3^ copies/µL). Similarly, six avian influenza virus genes were also simultaneously detected. The sensitivity to detecting HBV DNA was enhanced extraordinarily from the pM to the fM range. Bacteria could be detected even at < 10 CFU in leafy greens. Additionally, seven antibiotic resistance genes were identified in various cell lysates and *E. coli* strains.

The use of the protein microarray identified six biomarkers for hepatocellular carcinoma and four biomarkers with a potential to differentiate between active and latent tuberculosis. The identification of 85 deferentially expressed plasma proteins provided insights in understanding the mechanism of the yang-deficiency constitution. The lectin microarrays proved their strength in the profiling of glycosylation patterns. A strategy of bioinformatics analysis in conjunction with a protein microarray shortlisted 27 drug-binding protein targets. The study of the antifouling property, vaccine candidate screening in clinical trials, and the study of enzyme kinetic parameters were carried out using protein microarrays. During the COVID-19 pandemic, the existing knowledge of protein microarrays was exploited to develop SARS-CoV-2 variant microarrays to study vaccinated and nonvaccinated persons’ humoral immunity and antigen–antibody interactions. The microarray differentiated between COVID-19 and the influenza virus with a good sensitivity and specificity.

The peptide microarrays were explored to detect allergen peptide-specific antibodies, for the quantitative screening of the matrix metalloproteinase-2 levels in normal and four cancer cells, and for extracellular vesicle capture. One of the designed peptide microarrays identified a peptide that neutralizes RBD-ACE2 interacting antibodies in COVID-19 infected patients. Glycan microarrays were explored to estimate the IC50 values and to identify the secondary binding site in human macrophage galactose-type lectin, binding to eight salmonella isolates and seven diarrheagenic *E. coli* isolates. Both heparin and fucoidan showed specific recognition to the SARS-CoV-2 spike protein. These carbohydrates can be used to develop a cost-effective point-of-care diagnostic kit for SARS-CoV-2 identification. An antibody microarray was used to screen for COVID-19 antigens in patient sera, for coronary artery stenosis serum protein profiling, to investigate the differentially expressed proteins in human sera administered with three varying traditional medicinal constitutions. The use of an antibody microarray identified a new biomarker for primary colorectal cancer tissue. Similarly, several interesting biomarkers were identified for coronary artery stenosis and schizophrenia. The next studies should focus on further scrutinizing the potential of the identified biomarkers in those disease diagnoses. In the case of a COVID-19 sample analysis, the MosaiQ based microarray showed an 88% sensitivity, which was >10% higher than that of the commercial competitors. Aptamers were only explored for toxin and organophosphorus pesticide detection studies. The obtained detection levels were highly satisfactory, with picogram and nanogram levels.

Apart from high-throughput screening, the sensitivity and specificity of the microarrays in the presence of interfering molecules are two important aspects to look into if one has to adopt them for their application. As described in the above paragraphs of various biomolecule-based microarrays, the sensitivity of some of the assays were in the range of the pictogram to nanogram level and were significantly similar to the existing methods. In the case of microarray specificity, the obtained results are highly satisfying considering their performance in identifying the protein biomarkers or antigens in various clinical samples (cancerous cells, tuberculosis samples, COVID-19 patient samples, and bacteria samples).

Over the next 5 years, we believe that the trend of using biomolecule-based microarrays for the existing applications will further flourish. In terms of fabrication strategies and biomolecule immobilization, only the existing methods will be studied. The GLAD strategy may be exploited further and explored for other biomolecule (protein, peptide, antibody, and glycan)-based microarrays. New macroporous polymers might be introduced as thin film layers to enhance biomolecule loading onto microarray chips. Additionally, various branched molecules (dendrimers) or nanoparticles with a high surface area and with reactive sites are potential molecules that can be explored. Similarly, the molecular imprinting approach to create target analyte recognition sites could be another potential possibility. During the last two decades, molecular imprinting has been one of the trending technologies in the field of separation and sensing studies. A variety of polymers or small molecules that can interact with the target molecule via various interactions (hydrogen bond interactions, Van der Waals interactions, ionic interactions, and electrostatic interactions) could be used to create high surface materials on the microarrays. The removal of the target molecule from the imprinted polymer material will result in cavities that have a specific affinity to the imprinted analyte only. In this study, we have observed that label-based detection is the most widely used method. We think, in the near future as well, that this trend will remain unaffected. Considering that there are several multidisciplinary on-going projects around the globe and considering the pandemic situation, researchers may further explore the potential of microarray platforms for COVID-19 studies. Two of the most likely potential future applications of microarrays could be in the fields of personalized medicine and vaccine candidate screening. Similarly, other areas where microarrays can be exploited are toxin screening and posttranslational modifications.

## Data Availability

Not applicable.
